# The clinical efficacy of melatonin in the treatment of patients with COVID-19: a systematic review and meta-analysis of randomized controlled trials

**DOI:** 10.3389/fmed.2023.1171294

**Published:** 2023-04-25

**Authors:** Po-Yu Huang, Jheng-Yan Wu, Ting-Hui Liu, Ya-Wen Tsai, Po-Tsang Chen, Chia-Te Liao, Han Siong Toh

**Affiliations:** ^1^Department of Internal Medicine, Chi Mei Medical Center, Tainan, Taiwan; ^2^Department of Nutrition, Chi Mei Medical Center, Tainan, Taiwan; ^3^College of Medicine, Graduate Institute of Medicine, Kaohsiung Medical University, Kaohsiung, Taiwan; ^4^Department of General Internal Medicine, Chi Mei Medical Center, Tainan, Taiwan; ^5^Center of Integrative Medicine, Chi Mei Medical Center, Tainan, Taiwan; ^6^Division of Endocrinology and Metabolism, Department of Internal Medicine, Chi Mei Medical Center, Tainan, Taiwan; ^7^Department of Public Health, College of Medicine, National Cheng Kung University, Tainan, Taiwan; ^8^Division of Cardiology, Department of Internal Medicine, Chi Mei Medical Center, Tainan, Taiwan; ^9^Department of Intensive Care Medicine, Chi Mei Medical Center, Tainan, Taiwan; ^10^Institute of Clinical Medicine, College of Medicine, National Cheng Kung University, Tainan, Taiwan; ^11^Department of Health and Nutrition, Chia Nan University of Pharmacy and Science, Tainan, Taiwan

**Keywords:** COVID-19, melatonin, SARS-CoV-2, systematic review, meta-analysis

## Abstract

**Background:**

The COVID-19 pandemic has resulted in significant morbidity and mortality worldwide, with cytokine storm leading to exaggerating immune response, multi-organ dysfunction and death. Melatonin has been shown to have anti-inflammatory and immunomodulatory effects and its effect on COVID-19 clinical outcomes is controversial. This study aimed to conduct a meta-analysis to evaluate the impact of melatonin on COVID-19 patients.

**Methods:**

PubMed, Embase, and Cochrane Central Register of Controlled Trials were searched without any language or publication year limitations from inception to 15 Nov 2022. Randomized controlled trials (RCTs) using melatonin as therapy in COVID-19 patients were included. The primary outcome was mortality, and the secondary outcomes included were the recovery rate of clinical symptoms, changes in the inflammatory markers like C-reactive protein (CRP), erythrocyte sedimentation rate (ESR), and neutrophil to lymphocyte ratio (NLR). A random-effects model was applied for meta-analyses, and further subgroup and sensitivity analyses were also conducted.

**Results:**

A total of nine RCTs with 718 subjects were included. Five studies using melatonin with the primary outcome were analyzed, and the pooled results showed no significant difference in mortality between melatonin and control groups with high heterogeneity across studies identified (risk ratio [RR] 0.72, 95% confidence interval [CI] 0.47–1.11, *p* = 0.14, *I^2^* = 82%). However, subgroup analyses revealed statistically significant effects in patients aged under 55 years (RR 0.71, 95% CI 0.62–0.82, *p* < 0.01) and in patients treated for more than 10 days (RR 0.07, 95% CI 0.01–0.53, *p* = 0.01). The recovery rate of clinical symptoms and changes in CRP, ESR, and NLR were not statistically significant. No serious adverse effects were reported from melatonin use.

**Conclusion:**

In conclusion, based on low certainty of evidence, the study concluded that melatonin therapy does not significantly reduce mortality in COVID-19 patients, but there are possible benefits in patients under 55 years or treated for more than 10 days. With a very low certainty of evidence, we found no significant difference in the recovery rate of COVID-19 related symptoms or inflammatory markers in current studies. Further studies with larger sample sizes are warranted to determine the possible efficacy of melatonin on COVID-19 patients.

**Systematic review registration:**

https://www.crd.york.ac.uk/prospero/, identifier CRD42022351424.

## Introduction

As of November 29, 2022, there have been 641,883,458 confirmed cases of Coronavirus disease 2019 (COVID-19) reported to World Health Organization (1). Although most patients with severe acute respiratory syndrome coronavirus 2 (SARS-CoV-2) infection present with mild-to-moderate COVID-19, a significant portion may progress to severe-to-critical disease and even death (2, 3). The main cause of severe COVID-19 is dysregulated inflammations and cytokine storm (4). Therefore, controlling the exaggerated immune response and preventing associated multi-organ dysfunction and death has become a critical issue during the COVID-19 pandemic. Although systemic corticosteroid and interleukin-6 (IL-6) blockade had been shown to be clinically effective in lowering the mortality of patients with severe-to-critical COVID-19 (5, 6), their uses may be associated with adverse effect such as immunosuppression and secondary infection. An available and more tolerable anti-inflammatory agent against COVID-19 is therefore urgently needed.

Melatonin, a hormone produced by the pineal gland, has been found to have anti-inflammatory and immunomodulation effects, and has been repurposed as a potential therapy for patients with SARS-CoV-2 infection (7–9). A recent meta-analysis showed that melatonin use can effectively reduce inflammatory markers such as tumor necrosis factor (TNF)-α and IL-6 level (10). Two meta-analyses have also reported the clinical benefits of melatonin, including improved recovery rate of symptoms, in treating patients with COVID-19 (11, 12). However, these findings were based on small numbers of randomized controlled trials (RCTs). Recently, more RCTs with larger sample sizes have been published, but with opposing results and findings (13–15). To resolve this conflicting issue, we conducted this systematic review and meta-analysis of RCTs to provide reliable and updated results on the effect of melatonin in treating patients with COVID-19.

## Methods

### Inclusion and exclusion criteria

Studies were included in the qualitative analysis if they fulfilled the criteria listed below: (1) randomized controlled trials (RCTs), (2) adult participants (age 18 and above) diagnosed with COVID-19, and (3) use of melatonin as a therapeutic intervention. Exclusion criteria included letters, study protocols, phase I or II studies, case reports, animal studies, duplicated publications, unrelated studies, and literatures with ineligible outcomes. These studies were removed by screening the titles and abstracts, and the full texts of the remainders were obtained for quality assessment and data synthesis. Further, individual journals and conference proceedings, reference lists of related studies, systematic reviews, and meta-analyses were manually examined to identify any additional relevant publications.

### Search strategy

The publication search was conducted systematically on November 15, 2022 in three bibliographic databases: PubMed, Embase, and Cochrane Central Register of Controlled Trials, without limitations on publication year, publication status or language. Keywords were selected from a combination of controlled vocabulary and free-text terms, and were input for literature searching guided by Boolean operators (Supplementary Table 1). The study protocol was registered in PROSPERO (CRD42022351424) and the study was reported in accordance with the Preferred Reporting Items for Systematic Review and Meta-analysis (PRISMA) 2020 statement guidelines (16).

### Data extraction and study quality assessments

We extracted baseline characteristics and outcomes of the included studies. The risk of bias in the included RCTs was assessed using the Cochrane Risk-of-Bias (RoB) tool 1.0 (17). The assessment considered six domains of bias (selection, performance, detection, attrition, reporting, and other bias) and reported as low, unclear, or high risk of bias. A study with a low risk of bias in all key domains was considered high quality.

Data extraction and risk of bias assessment were performed by two independent reviewers (THL and PYH) for each selected study. The certainty of evidence for each outcome was assessed independently by the two authors based on the Grading of Recommendations Assessment, Development and Evaluation (GRADE) framework (18). Any disagreements among reviewers were discussed with a third reviewer (JYW) until a consensus was reached.

### Outcomes

The primary outcome of this study was mortality rate, and secondary outcomes were recovery rate of clinical symptoms (e.g., fever, chest pain, and dyspnea) and changes in the inflammatory markers, including C-reactive protein (CRP), erythrocyte sedimentation rate (ESR), and neutrophil to lymphocyte ratio (NLR). Additionally, data on the adverse effects of melatonin usage was also collected for safety profiling.

### Statistical analysis

We conducted a meta-analysis using the Mantel-Haenszel and inverse variance-weighted random-effect models to estimate the overall pooled effect (19, 20). For dichotomous data, the effect size was expressed as the relative risk (RR) with a 95% confidence interval (95% CI); for continuous data, it was presented as the mean differences (MD) with a 95% CI. To account for inter-patient variability in continuous data, we calculated the change values from baseline to the end of the follow-up period using the baseline and end-of-study values and their associated standard deviations (SD), using a correlation coefficient of 0.5 if the change values were not reported (19). When continuous outcomes were reported as median, range, and interquartile range, we estimated the means and SD using the formula described by Hozo et al. (21).

Between-trial heterogeneity was assessed using the Cochran’s Q and Hedge’s I^2^ statistic tests, and was interpreted as low level of heterogeneity when *I*^2^ ≤ 25%, moderate when 25% < I^2^ < 75%, and high when *I*^2^ ≥ 75% (22, 23). For meta-analyses with at least ten studies, we evaluated the potential publication bias by visual assessment of funnel plot asymmetry. However, when included studies are less than ten, the funnel plot would not be performed due to its unreliability.

Besides, trial sequential analysis (TSA) was conducted to control types I and II errors and prevent multiplicity phenomenon (24). The analysis was performed on the basis of the relative risk reduction of 20%, pre-specified type I error of 5%, and type II error of 20% (power at 80%). No further trials are warranted if the cumulative Z curve crossed the trial sequential monitoring boundary or required information size boundary, which proved a robust result.

All *p*-values were two-tailed, with a significance level set at 0.05, except for the statistical tests for heterogeneity, which used a significance level of 0.10. All analyses were performed using Review Manager software version 5.4 (Nordic Cochrane Centre, Cochrane Collaboration) and STATA (Version 16, Stata Corp., 2019, College Station, TX, Stata Corp., LP).

### Subgroup analyses and sensitivity analyses

When moderate to high heterogeneity is detected among trials, subgroup and sensitivity analyses are conducted (19). After specialist consultation and literature reviewing, subgroup analyses are conducted to examine the causes of heterogeneity in the primary outcome based on treatment duration, dosage of melatonin, age, and severity of disease. An age cutoff of 55 was chosen based on previous studies on melatonin use for sleep disorder (25). Melatonin dose lower than 10 mg/day was referred as low dose, and high dose is greater than or equal to 10 mg/day (26). A significance test for heterogeneity across subgroup is performed according to the Cochrane handbook for Systematic Reviews of Interventions to investigate differences between subgroups, as random-effect model was applied throughout our analyses (19). Sensitivity analyses evaluated the individual study’s influence on the overall estimates by removing one article at a time and pooling data from the remaining studies.

## Results

### Description of included studies

**FIGURE 1:**
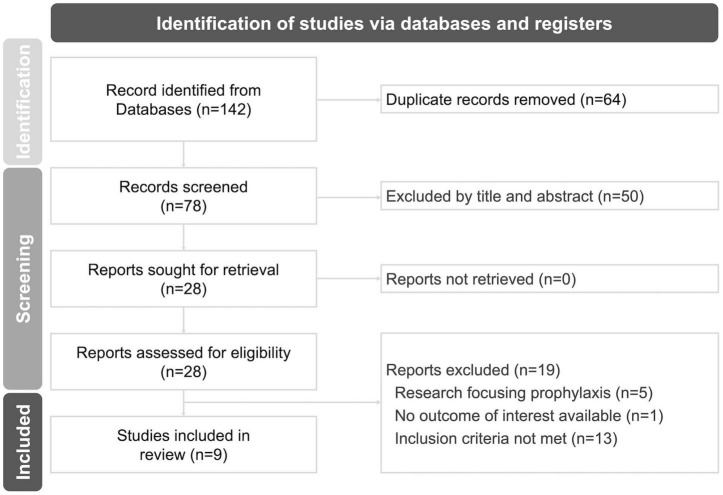
Meta-analysis flow-chart diagram according to the PRISMA guideline.

The flowchart illustrating the research selection process is presented in Figure 1. A total of 140 records were screened during the electronic database searching process, and two additional studies were retrieved by screening reference lists of related articles. Eventually, nine RCTs involving 718 subjects were identified and included in the final meta-analyses (13–15, 27–32). In the experimental group, the intervention consisted of melatonin plus standard care, while in the control group, nearly all comparators were standard care alone (15, 27–32), except for two studies that included a matched placebo (13, 14) (Supplementary Table 2).

The main characteristics of the enrolled studies are listed in Table 1. Of the included studies, two were conducted by Hasan et al. (15, 30) and shared patient characteristics. Although the reported numbers of randomized patients and focused outcomes differed in the two articles, we considered the results to be derived from the same population and counted the enrolled subjects only once to avoid duplication. All studies enrolled hospitalized patients except for the study by Fogleman et al. (14), which included only outpatients. Among the five studies eligible for primary outcome analysis (13, 15, 29, 31, 32), the duration of treatment was more than 10 days in two studies (15, 29), and less than 10 days in three studies (13, 31, 32). Melatonin was administered at night-time in all studies, and a high dose melatonin was utilized in three studies (13, 15, 32). The average age of the patients was above 55 in two studies (13, 15), and below 55 in the other three studies (29, 31, 32). The severity of COVID-19 in the enrolled patients was severe in three studies (13, 15, 32).

**TABLE 1:** Summary of the baseline characteristics of the included studies.

References	Country	Sample size (*n*)	Male (%)	Age, years [mean ± SD or median (IQR)]	Severity of disease	Melatonin dose and regimen	Control group	Treatment dose	Treatment duration (days)	Primary outcome
Alizadeh et al. (27)	Iran	M: 14 C: 17	M: 64.3 C: 47.1	M: 37.6 ± 8.2 C: 34.5 ± 8.2	Mild to moderate	Melatonin 6 mg/day for 2 weeks, consumed half an hour before bedtime every night in low light conditions	NR	6 mg/day	14	Inflammatory markers
Alizadeh et al. (13)	Iran	M: 33 C: 34	M: 57.6 C: 70.6	M: 61.3 ± 18.1 C: 65.4 ± 19.3	Severe (COVID-19 admitted to the ICU and had undergone invasive ventilation)	Melatonin 21 mg/day for 5 nights via NG tube	Matched placebo	21 mg/day	5	Mortality
Ameri et al. (32)	Iran	M: 109 C: 117	M: 43.1 C: 41.9	M: 54.60 ± 11.51 C: 54.69 ± 13.40	Severe	Melatonin 10 mg/day for 7 days	NR	10 mg/day	7	Mortality
Darban et al. (28)	Iran	M: 10 C: 10	NR	All: 59 ± 19	Severe (who admitted to ICU)	Melatonin 24 mg/day for 10 days	NR	24 mg/day	10	Changes in severity of hypoxemia
Farnoosh et al. (29)	Iran	M: 24 C: 20	M: 58.3 C: 60.0	M: 50.8 ± 14.4 C: 53.0 ± 14.1	Mild to severe	Melatonin 9 mg/day for 14 days	NR	9 mg/day	14	Symptoms and laboratory parameters
Fogleman et al. (14)	U.S.	M: 32 C: 34	M: 34.4 C: 29.4	M: 52 (14) C: 54 (11)	Mild (patients were excluded if they were hospitalized)	Melatonin 10 mg/day at bedtime for 14 days	Cornstarch	9 mg/day	14	Symptoms
Hasan et al. (15, 30) (Hasan-A, Hasan-B)	Iraq	M: 82 C: 76	M: 70.7 C: 73.7	All: 56.3 ± 7.7 M: 56.8 ± 7.5 C: 55.7 ± 8.0	Severe	Melatonin 10 mg/day for 14 days, 20 - 30 min before bedtime	NR	10 mg/day	14	Thrombosis/ inflammatory markers
Mousavi et al. (31)	Iran	M: 48 C: 48	M: 52.1 C: 37.5	M: 51.1 ± 15.9 C: 54.8 ± 15.3	Moderate to severe (hospitalized)	Melatonin 3 mg/day for 7 days, 1 h before bedtime	NR	3 mg/day	7	Sleep quality

C, control group; ICU, intensive care unit; M, Melatonin group; NG, nasogastric; NR, not reported; SD, standard deviations; U.S, United States.

### Risk of bias assessment and certainty of evidence (GRADE)

**FIGURE 2:**
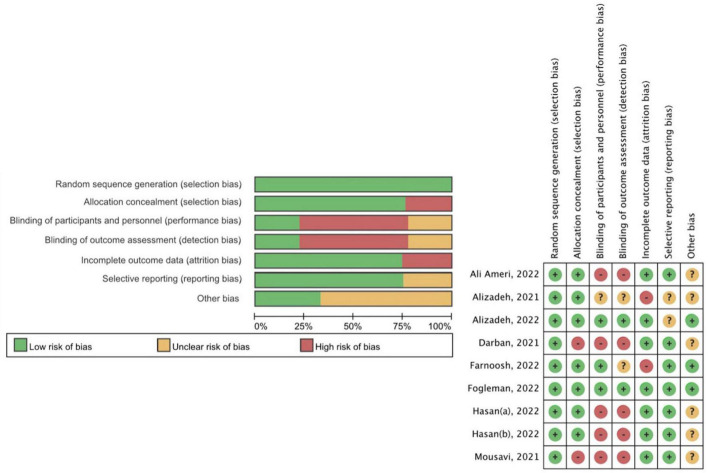
Methodological quality assessment of the included studies (Cochrane risk-of-bias tool 1.0).

None of the studies was excluded based on quality according to the Cochrane RoB tool 1.0 (Figure 2). Studies containing one or more domains with high risk of bias would be classified as “high risk of overall bias.” Seven studies demonstrated a high risk of overall bias (15, 27–32), while one showed unclear risk of overall bias (13), and one study presented low risk of bias in all domains (14). The failure in the blinding process resulted in the performance bias and detection bias, which is the main source of bias in those studies with an overall high risk of bias (13, 15, 29–31). For example, the loss of allocation concealment and blinding process in Darban et al. (28) constituted a significant risk of bias in two relevant domains.

Regarding the certainty of evidence, the primary outcome was judged to be low-quality evidence (Supplementary Figure 1). Other secondary outcomes were judged to be very low-quality evidence due to the serious risk of bias and imprecision of the outcome measurement.

### Primary and secondary outcome

**FIGURE 3:**
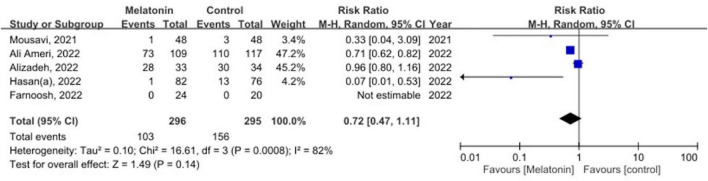
The forest plot depicts the mortality by comparing the melatonin group versus control group.

Five studies with the primary outcome comprising 547 patients were analyzed (13, 15, 29, 31, 32). Our meta-analysis found that the administration of melatonin did not significantly reduce the mortality rate, with high heterogeneity across studies identified (RR 0.72, 95% CI 0.47–1.11, *p* = 0.14; *I*^2^ 82%, *p* for heterogeneity < 0.01) (Figure 3).

**FIGURE 4:**
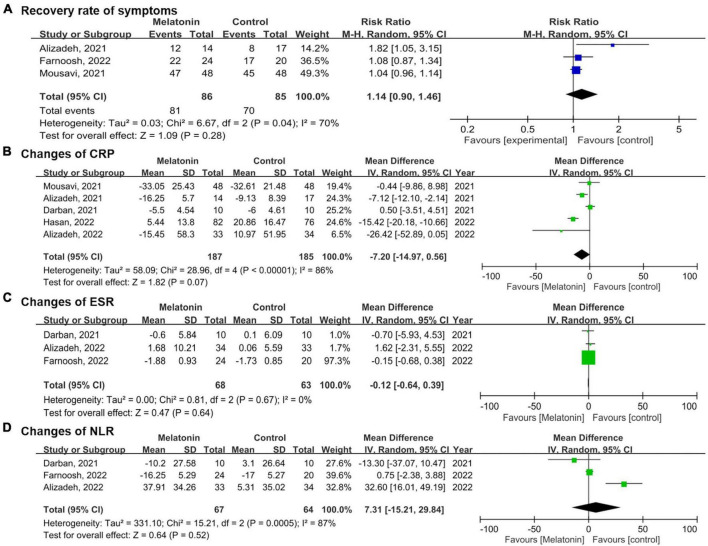
Forest plots demonstrate the secondary outcomes comparing the melatonin group versus control group in **(A)** recovery rate of symptoms, **(B)** changes of C-reactive protein (CRP), **(C)** changes of Erythrocyte sedimentation rate (ESR), **(D)** changes of Neutrophil-to-lymphocyte ratio (NLR).

As for the secondary outcomes, the pooled results showed no significant difference in the recovery rate of symptoms between the melatonin group and the control group (RR 1.14, 95% CI 0.90–1.46, *p* = 0.28; *I*^2^ 70%, *p* for heterogeneity = 0.04) (Figure 4) (27, 29, 31). Concerning the changes in CRP, no significant difference was identified between the two groups as well after pooling data from five studies (MD −0.12, 95% CI −0.64 to 0.39, *p* = 0.64; I2 0%, p for heterogeneity = 0.67) (13, 27, 28, 30, 31). No statistically significant effects were revealed between the groups regarding changes in ESR (MD −0.12, 95% CI −0.64 to 0.39, *p* = 0.64; *I*^2^ 0%, *p* for heterogeneity = 0.67) and NLR (MD 7.31, 95% CI −15.21 to 29.84, *p* = 0.52; *I*^2^ 87%, *p* for heterogeneity < 0.01) after melatonin administration (13, 28, 29).

**FIGURE 5:**
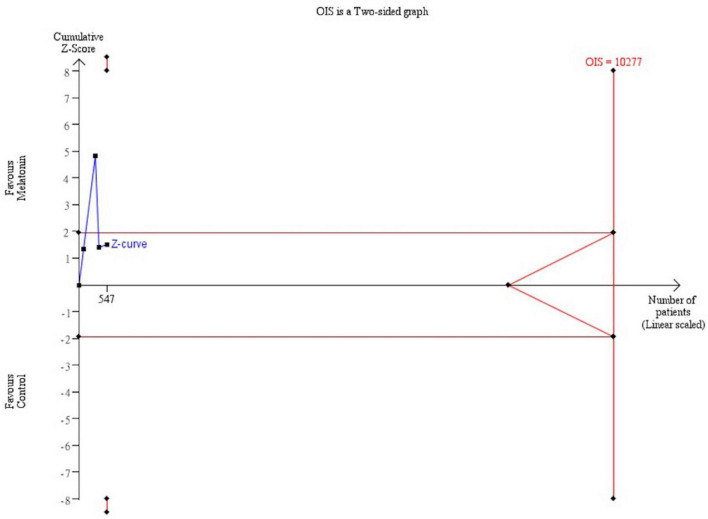
Trial sequential analysis of mortality rate. A diversity adjusted information size of 10,277 patients was calculated using 5% of type 1 error (2-sided), a power of 80%, an anticipated relative risk of 20.0%.

**FIGURE 6:**
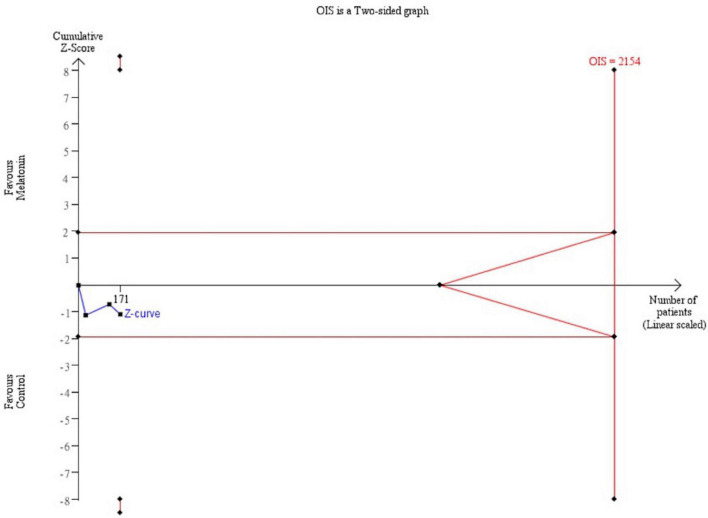
Trial sequential analysis of recovery rate of symptoms. A diversity adjusted information size of 2,154 patients was calculated using 5% of type 1 error (2-sided), a power of 80%, an anticipated relative risk of 20.0%.

Trial sequential analysis was performed for the mortality rate and recovery rate of symptoms. Regarding the primary outcome, TSA indicated that the Z-curve did not cross the traditional boundary, and only 5.3% of the optimal sample size (547/10277 patients) was accrued in the current analysis (Figure 5). For the recovery rate of symptoms, TSA also failed to reach the traditional boundary and the optimal sample size (171/2154 patients) (Figure 6).

### Adverse events

Most of the included studies did not mention any specific adverse events associated with the use of melatonin. However, Mousavi et al. (31) noted that no severe side effects were reported with a dosage of 3 mg for older adults, although the optimal dose had not yet been determined. The only reported side effects were nausea, vomiting, and headache. In contrast, Ameri et al. (32) found more adverse events in the control group than in the melatonin group. Dizziness was the most prevalent side effect in the melatonin group, while stroke, gastrointestinal hemorrhage, and hematuria were found in the control group.

### Subgroup analyses

Due to the heterogeneity of the results, further subgroup analyses were conducted in the meta-analysis on the mortality rate (Table 2). In patients under 55 years of age, melatonin significantly reduced the mortality rate (RR 0.71, 95% CI 0.62 to 0.82, *p* < 0.01), while no significant effect was observed in patients over 55 years old (RR 0.28, 95% CI 0.00–18.96, *p* = 0.56) (Supplementary Figure 2).

**TABLE 2:** Summary of subgroup analyses for primary outcome.

Subgroups	No. of studies	No. of patients	RR	95% CI	*I* ^2^	*p*-value for subgroup difference
**Age**						0.67
≥55 years	2	225	0.28	0.00–18.96	94%	
<55 years	3	591	0.71	0.62–0.82	0%	
**Treatment duration**						0.02
≥10 days	2	202	0.07	0.01–0.53	–	
<10 days	3	389	0.81	0.60–1.09	73%	
**Treatment dose**						0.49
≥10 mg/day	3	451	0.74	0.48–1.15	87%	
<10 mg/day	2	140	0.33	0.04–3.09	–	
**Severity**						
Severe COVID-19	3	451	0.74	0.48–1.15	87%	

The results showed a statistically significant difference in mortality rates between the groups in patients who received treatment for more than 10 days, with melatonin demonstrating a lower mortality rate than placebo (RR 0.07, 95% CI 0.01–0.53, *p* = 0.01) (Supplementary Figure 3). However, there was no significant difference observed in patients who received treatment for less than 10 days (RR 0.81, 95% CI 0.60–1.09, *p* = 0.16). Additionally, a significant difference was found in the mortality rate among the different subgroups based on the duration of treatment (*p* = 0.02). Moreover, it has not been observed that either high or low doses of melatonin are associated with a reduction in mortality (Supplementary Figure 4).

Regarding disease severity, there was no significant difference in the subgroup of severe COVID-19 (RR 0.74, 95% CI 0.48–1.15) (Supplementary Figure 5). The studies regarding mild to moderate disease did not recorded mortality and thus, the data was not available.

### Sensitivity analyses

We performed sensitivity analyses by systematically excluding each study one by one and using the total mortality rate as the outcome (Supplementary Table 3). The aim was to determine if any single study was responsible for the significant results observed. After excluding either the study by Ameri et al. (32) or Alizadeh et al. (13), the pooled relative risk decreased significantly from 0.72 to 0.31. However, we did not observe any noticeable decrease in heterogeneity during the one-by-one exclusion process.

### Publication bias

As there were only five studies included in the analysis for the primary outcome, it was not possible to perform a funnel plot to detect potential publication bias, as the small number of studies makes it difficult to identify any asymmetry.

## Discussion

At the beginning of the COVID-19 epidemics, melatonin was discovered to interact with the viral main protease and its receptor, angiotensin-converting enzyme 2, on the cell surface (33). Besides, melatonin was also found to convert proinflammatory M1 macrophages to the M2 phenotype macrophages, and hence, decrease inflammatory reaction during SARS-CoV-2 infection (34). Despite its possible protective role due to its anti-inflammatory, anti-oxidant, and immunomodulatory action, further clinical studies are in need to provide more evidence on its effect in the prevention and treatment of COVID-19 (35).

Based on this updated meta-analysis included 9 RCTs with 718 patients, we found that melatonin may not offer additional benefits to patients with COVID-19 in general, with regards to mortality, recovery of the symptoms, and the reduction in inflammatory markers such as CRP, ESR, and NLR. However, there is evidence that certain patient groups, including those aged under 55 years or with a longer treatment duration, may benefit from melatonin therapy. Still, further large-scale RCT are required to explore the optimal dose and duration of melatonin and its beneficial effect of melatonin in certain patient subgroup due to the low certainty of evidence of current available studies.

In the subgroup analysis, the melatonin group showed a significantly lower mortality rate than the control group in patients aged under 55 years. Previous studies have shown that melatonin production decreases with age (36–38), especially in patients aged 55 and older who experience poor sleep quality compared to healthy older adults without sleep complaints (25, 39). In light of this, a subgroup analysis was conducted with an age cut-off of 55, which revealed that only patients under 55 years of age experienced a mortality benefit from melatonin therapy. This finding might suggest that higher doses of exogenous melatonin may be necessary in older patients to achieve clinical efficacy, as natural melatonin production is lower in this population.

On the other hand, those who received melatonin for ten or more days was found to have lower mortality rate in our subgroup analysis. In fact, the optimal dosage and duration of melatonin as an anti-inflammatory and antioxidant agent remain uncertain (26, 35). There are two recognized phases in the pathogenesis of COVID-19, and severe cases with mortality is associated with the later phase of hyperinflammation and rampant release of cytokines (40). Anti-inflammatory agents, such as steroids, are preferred for their greater efficacy during the late stages of the disease. Prolonged prescription of these agents may provide additional benefits (41). The role of melatonin during the two phases of COVID-19 pathogenesis is also differs, with evidence suggesting that it may play a pro-inflammatory role during the early stages of inflammation and an anti-inflammatory role in the later stages (8, 9). These findings might indicate that prolonged administration of melatonin may benefit COVID-19 patients, as it has a preferable anti-inflammatory effect during the late stage of infection. Moreover, whether high-dose melatonin can be more effective or harmful due to its side effects is still a subject of debate (26). In our study, high dose melatonin was not found to have beneficial effect but two of the studies prescribed melatonin with a shorter period (13, 32). However, these results should be interpreted cautiously due to the limited patient numbers in the studies, and additional trials are necessary to determine the appropriate dosage and duration of melatonin to achieve efficacy in diverse populations.

Of note, the findings of the present meta-analysis differ from those of previous meta-analyses on clinical recovery (11, 12). In Lan et al.’s (11) study, the analysis of three RCTs showed that melatonin was associated with a significantly higher clinical recovery rate compared to the comparators (odds ratio [OR] 3.67; 95% CI 1.21 to 11.12; *I*^2^ 0%, *p* = 0.02). Similarly, in another meta-analysis included five RCTs and one retrospective cohort study by Wang et al. (12), the clinical recovery rate was also higher in subjects treated with melatonin (OR 3.05, 95% CI 1.47–6.31; *I*^2^ 0%, *p* = 0.003) than in the control group. With more evidence available, our findings were based on the analysis of nine RCTs with a larger number of patients compared to these two studies. Albeit the low heterogeneity and statistical significance in the two studies, we preferred the random-effect model with the expression of risk ratio in our study, compared with the fixed-effect model and odds ratio in the previous studies. The fixed-effect model can be easily biased and only provides unbiased estimates if treated and untreated individuals do not differ with respect to the trend in the outcome of interest over time (42). Therefore, we believe that the present study provides more updated and robust evidence on this issue.

Nevertheless, our study has several limitations. First, although it is the most recent systematic review investigating the usefulness of melatonin in patients with SARS-CoV-2 infection, the sample size remained small, and the TSA analysis was inconclusive. Therefore, further large-scale RCT is needed to provide more robust evidence regarding the treatment. Secondly, some findings in the present study were associated with high heterogeneity, which could be caused by the various dosage and treatment duration of melatonin, as well as the different course of the disease and the heterogeneous underlying diseases of the patients. Although we performed subgroup analysis to identify possible patient groups that might benefit from the treatment, there were still many other confounding factors that could not be identified due to the lack of associated data, such as specific underlying diseases. And the limited number of studies would be a concern while interpreting the result of the subgroup analyses. Third, our analyses could not provide analysis on safety profile due the limited data. Potential adverse effects, such as nausea, vomiting, and headache, could be observed at higher doses of melatonin, but no severe adverse event had been reported till date (32, 43).

## Conclusion

Based on the current available low certainty of evidence, our study found that melatonin did not decrease overall COVID-19 mortality, but subgroup analyses suggested potential benefits in patients under 55 years of age or those treated for over 10 days. With a very low certainty of evidence, no significant differences were observed in the recovery rates of relevant symptoms or inflammatory markers. Nevertheless, larger RCTs are necessary to confirm these findings and further elucidate the potential role of melatonin as an anti-inflammatory agent in the management of COVID-19.

## Data availability statement

The original contributions presented in this study are included in the article/Supplementary material, further inquiries can be directed to the corresponding author.

## Author contributions

P-YH, J-YW, T-HL, Y-WT, P-TC, and HT contributed to this study, including the conception and design of the research. P-YH and J-YW performed the data extraction and analyses and interpretation of the data. T-HL was consulted as a third reviewer in case of any disagreements. Y-WT provided assistance in statistics. P-TC ensured the quality of the study according to the PRISMA guideline. P-YH, J-YW, and T-HL drafted the manuscript. Y-WT, P-TC, HT, and C-TL revised the manuscript. All authors gave their final approval and agreed to all aspects of the work, ensuring its integrity and accuracy.
