# Supplementary material for: The clinical efficacy of melatonin in the treatment of patients with COVID-19: a systematic review and meta-analysis of randomized controlled trials
Source: Front Med (Lausanne). 2023 Apr 25;10:1171294. doi: 10.3389/fmed.2023.1171294 (PMC10166833; doi:10.3389/fmed.2023.1171294)
Supplement: Supplementary file 1 [file Data_Sheet_1.docx]

**Supplementary Data**

**Legends**

Table S1. Search Strategy.

Table S2. Standard care adopted in enrolled studies.

Table S3. Sensitivity analysis by excluding each study one-by-one.

Figure S1. GRADE assessment

Figure S2. The forest plot depicts subgroup of the mortality based on age comparing the melatonin group versus control group.

Figure S3. The forest plot depicts subgroup of the mortality based on treatment duration comparing the melatonin group versus control group.

Figure S4. The forest plot depicts subgroup of the mortality based on treatment dose comparing the melatonin group versus control group.

Figure S5. The forest plot depicts subgroup of the mortality based on severity comparing the melatonin group versus control group.

Table S1. Search Strategy

**Database: PubMed**

| No | Query | Results |
| --- | --- | --- |
| #1 | COVID-19 | 313,060 |
| #2 | SARS-CoV-2 Infection OR 2019 Novel Coronavirus Disease OR Coronavirus Disease 2019 OR Severe Acute Respiratory Syndrome Coronavirus 2 Infection | 301,210 |
| #4 | #1 OR #2 | 313,060 |
| #5 | Melatonin | 30,563 |
| #6 | Melatoni* | 30,584 |
| #7 | melatonergic agent | 152 |
| #8 | #5 OR #6 OR #7 | 30,637 |
| #9 | #8 AND #4 | 207 |
| #10 | (randomized controlled trial[pt] OR controlled clinical trial[pt] OR randomized[tiab] OR randomised[tiab] OR placebo[tiab] OR drug therapy[sh] OR randomly[tiab] OR trial[tiab] OR groups[tiab] NOT (animals [mh] NOT humans [mh])) | 4,893,717 |
| #11 | #9 AND #10 | 88 |

**Database: Cochrane Central Register of Controlled Trials**

| No | Query | Results |
| --- | --- | --- |
| #1 | Melatonin | 3565 |
| #2 | MeSH descriptor: [Melatonin] explode all trees | 1364 |
| #3 | #1 OR #2 | 3565 |
| #4 | COVID-19 | 13623 |
| #5 | MeSH descriptor: [COVID-19] explode all trees | 2553 |
| #6 | #4 OR #5 | 13623 |
| #7 | #3 AND #6 | 55 |

**Database: Embase**

| No | Query | Results |
| --- | --- | --- |
| #1 | **('coronavirus disease 2019'/exp OR '2019 novel coronavirus disease' OR '2019 novel coronavirus epidemic' OR '2019 novel coronavirus infection' OR '2019-ncov disease' OR '2019-ncov infection' OR 'covid' OR 'covid 19' OR 'covid 19 induced pneumonia' OR 'covid 2019' OR 'covid-10' OR 'covid-19' OR 'covid-19 induced pneumonia' OR 'covid-19 pneumonia' OR 'covid19' OR 'sars coronavirus 2 infection' OR 'sars coronavirus 2 pneumonia' OR 'sars-cov-2 disease' OR 'sars-cov-2 infection' OR 'sars-cov-2 pneumonia' OR 'sars-cov2 disease' OR 'sars-cov2 infection' OR 'sarscov2 disease' OR 'sarscov2 infection' OR 'wuhan coronavirus disease' OR 'wuhan coronavirus infection' OR 'coronavirus disease 2' OR 'coronavirus disease 2010' OR 'coronavirus disease 2019' OR 'coronavirus disease 2019 pneumonia' OR 'coronavirus disease-19' OR 'coronavirus infection 2019' OR 'ncov 2019 disease' OR 'ncov 2019 infection' OR 'new coronavirus pneumonia' OR 'novel coronavirus 2019 disease' OR 'novel coronavirus 2019 infection' OR 'novel coronavirus disease 2019' OR 'novel coronavirus infected pneumonia' OR 'novel coronavirus infection 2019' OR 'novel coronavirus pneumonia' OR 'paucisymptomatic coronavirus disease 2019' OR 'severe acute respiratory syndrome 2' OR 'severe acute respiratory syndrome 2 pneumonia' OR 'severe acute respiratory syndrome cov-2 infection' OR 'severe acute respiratory syndrome coronavirus 2 infection' OR 'severe acute respiratory syndrome coronavirus 2019 infection') AND ('melatonin'/exp OR 'melatonin' OR 'melatonina' OR 'melatonine' OR 'melatonite')** | 473 |
| #2 | **#1 AND [embase]/lim NOT ([embase]/lim AND [medline]/lim)** | 167 |
| #3 | **#1 AND [embase]/lim NOT ([embase]/lim AND [medline]/lim) AND ([randomized controlled trial]/lim OR 'controlled clinical trial'/de)** | 7 |

| Author | Standard care |
| --- | --- |
| Alizadeh, 2021 | Regular medications, and patients in both groups (if needed) received medications prescribed by physicians (hydroxychloroquine, acetaminophen, and naproxen) |
| Alizadeh, 2022 | Remdesivir 200 mg on the first day and 100 mg daily for 4 days thereafter, Corticosteroids, Anticoagulant (prophylactic dose), and sometimes Tocilizumab |
| Ameri, 2022 | Not reported |
| Darban, 2021 | Azithromycin 250 mg/day, Lopinavir/Ritonavir 100 mg/25 mg/day, Glucocorticoids, and necessary oxygen |
| Farnoosh, 2022 | Not reported |
| Fogleman, 2022 | Not reported |
| Hasan, 2022  (Hasan-A, Hasan-B) | Remdesivir 200 mg for day 1, and 100 mg for 4 days + Levofloxacin 500 mg/day + Dexamethasone 24 mg/day + Enoxaparin 6000 units once daily for prophylaxis and twice daily for therapeutic treatment of thrombosis |
| Mousavi, 2021 | Hydroxychloroquine, Atazanavir, methylprednisolone, azithromycin, naproxen, and Lopinavir/Ritonavir |

Table S2. Standard care adopted in enrolled studies (according to alphabetical order of the author’s name)

Table S3. Sensitivity analysis by excluding each study one-by-one

| Excluded Study | RR (95% CI) | I^2^ |
| --- | --- | --- |
| None | 0.72 (0.47 to 1.11) | 82 % |
| Mousavi, 2021 | 0.74 (0.48 to 1.15) | 87 % |
| Ameri, 2022 | 0.31 (0.02 to 4.99) | 90 % |
| Alizadeh, 2022 | 0.31 (0.06 to 1.69) | 74 % |
| Hasan-A, 2022 | 0.81 (0.60 to 1.09) | 73 % |
| Farnoosh, 2022 | 0.72 (0.47 to 1.11) | 82 % |


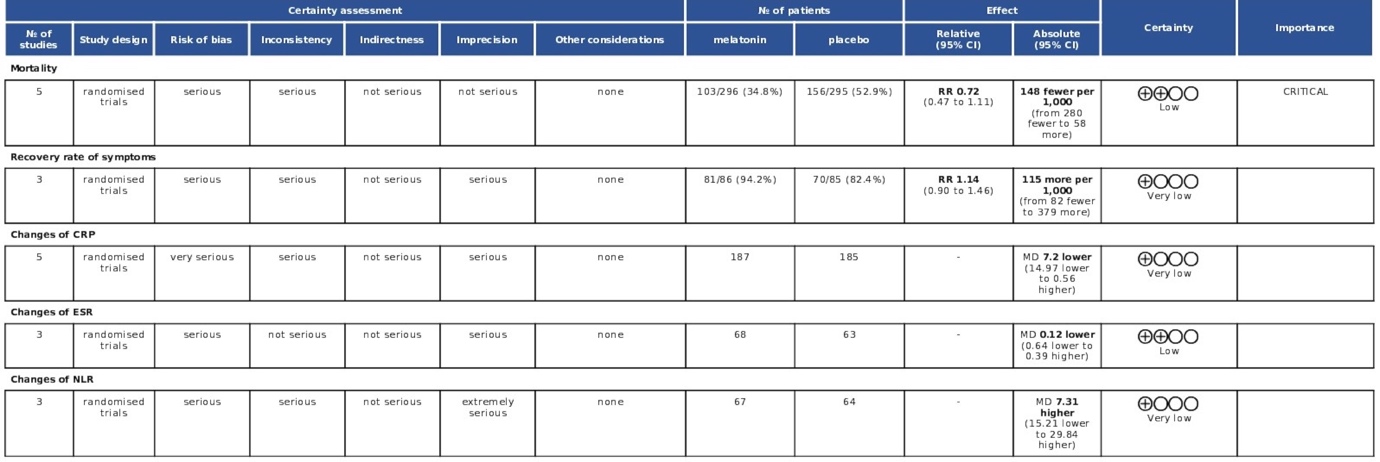


Figure S1. GRADE assessment


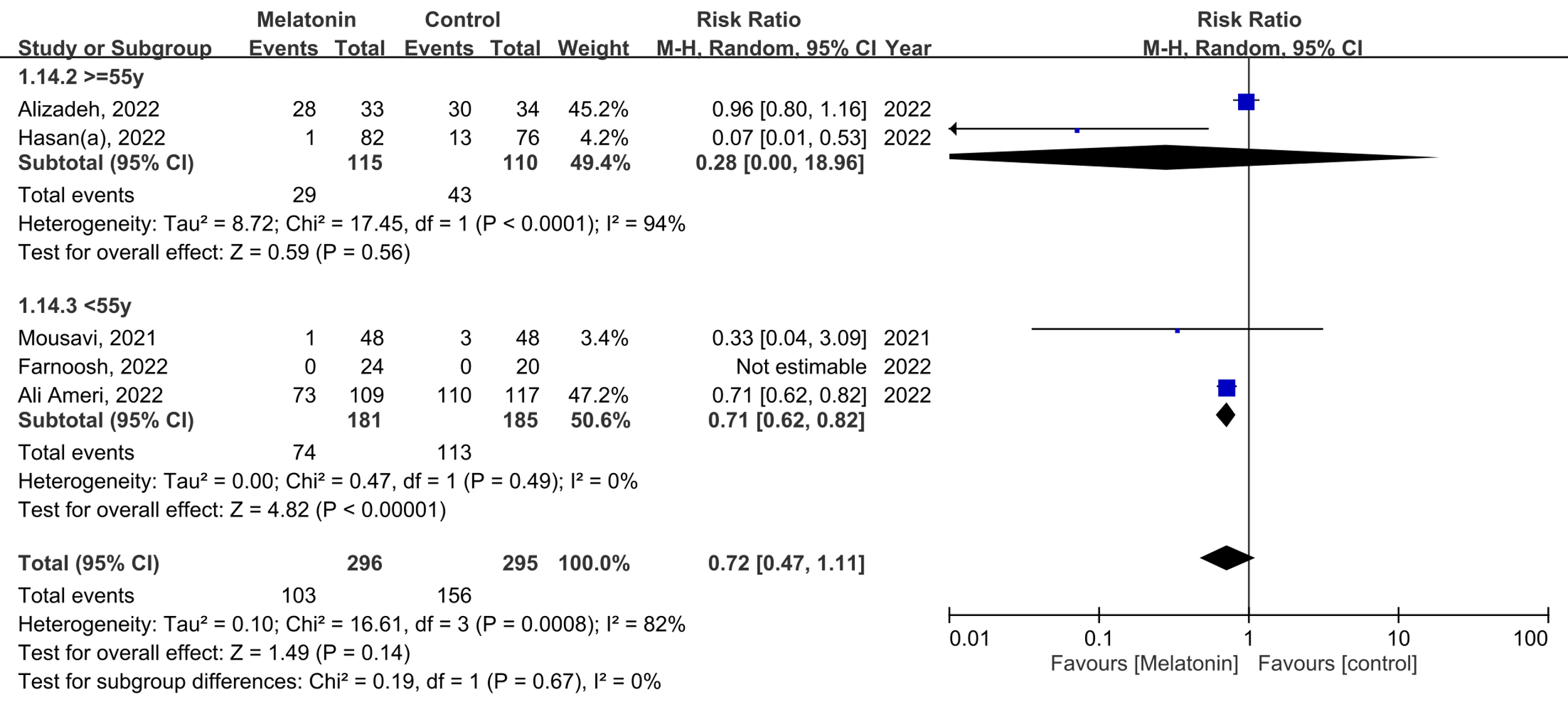


Figure S2. The forest plot depicts subgroup of the mortality based on age comparing the melatonin group versus control group.


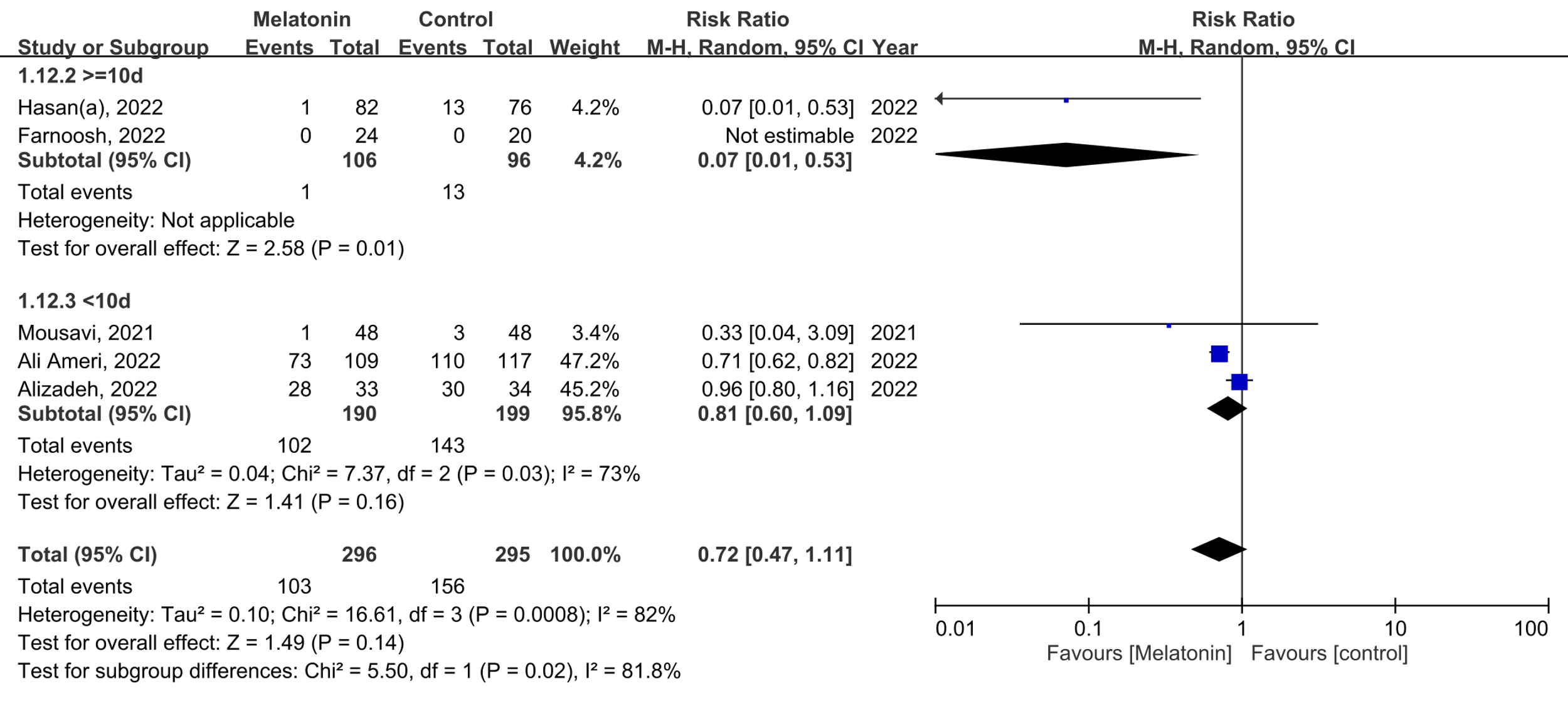


Figure S3. The forest plot depicts subgroup of the mortality based on treatment duration comparing the melatonin group versus control group.


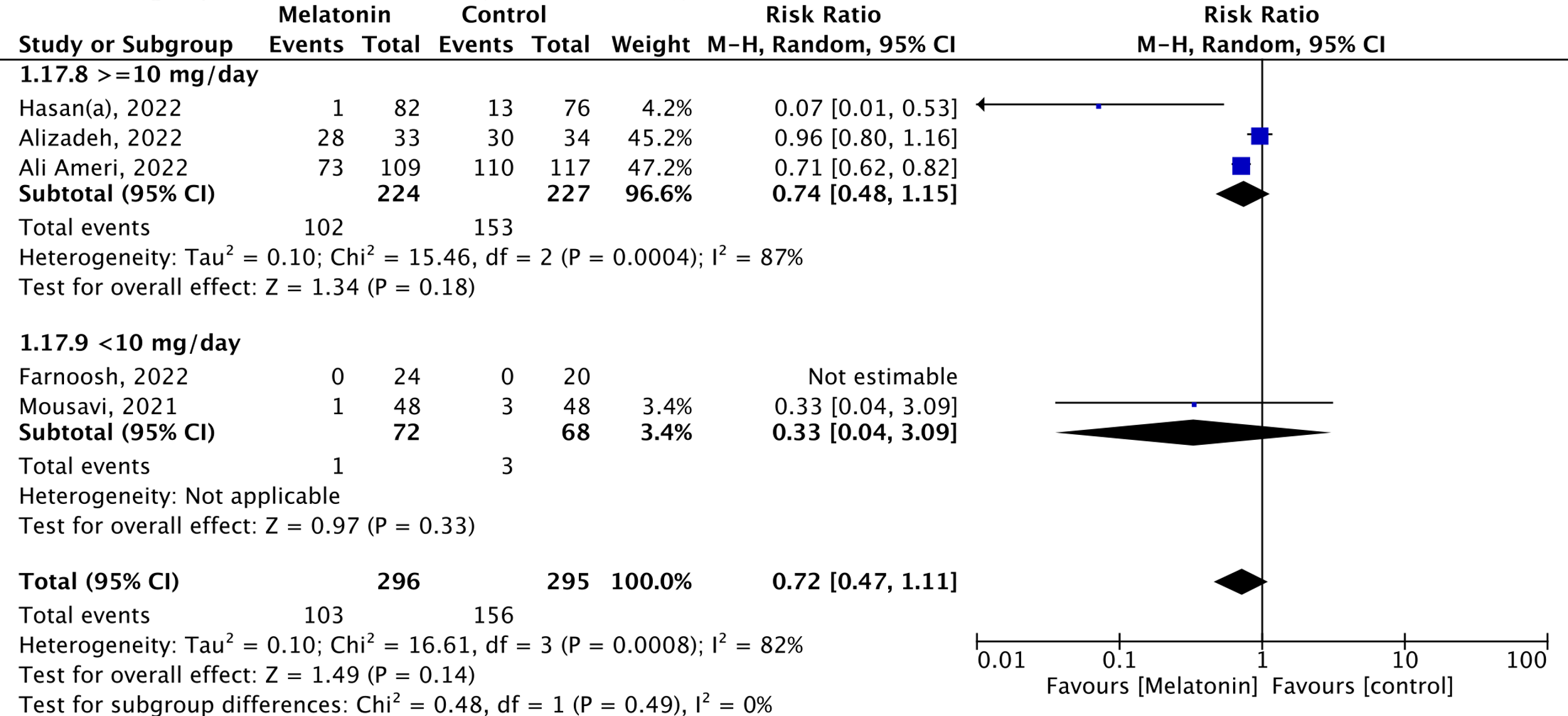


Figure S4. The forest plot depicts subgroup of the mortality based on treatment dose comparing the melatonin group versus control group.


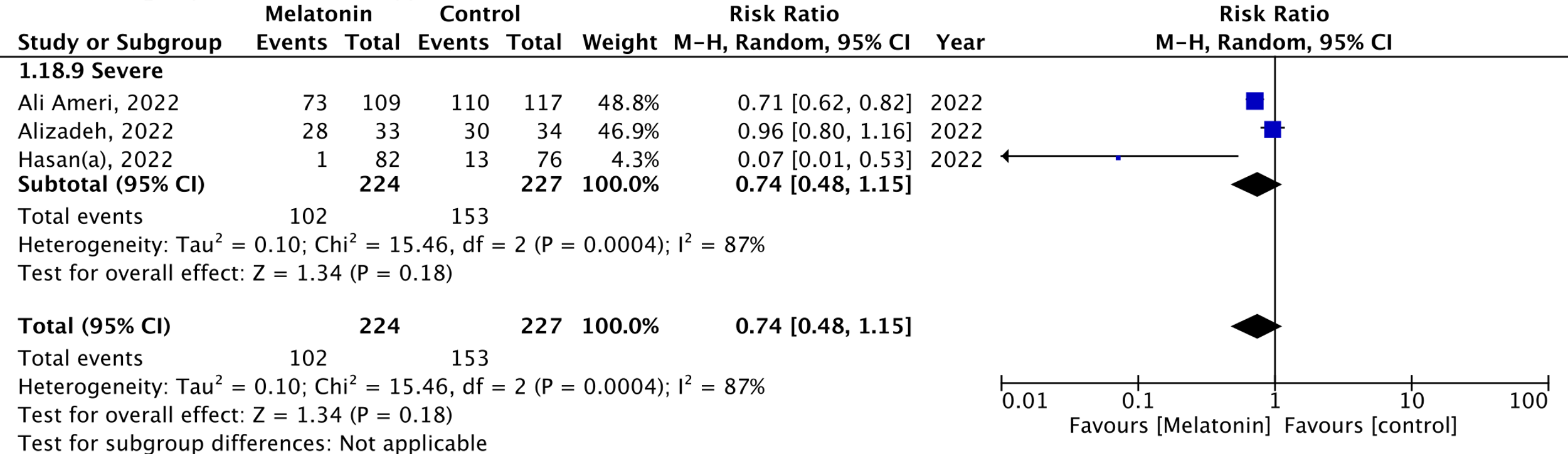


Figure S5. The forest plot depicts subgroup of the mortality based on severity comparing the melatonin group versus control group.
